# Model Selection for Non-Negative Tensor Factorization with Minimum Description Length

**DOI:** 10.3390/e21070632

**Published:** 2019-06-27

**Authors:** Yunhui Fu, Shin Matsushima, Kenji Yamanishi

**Affiliations:** 1 The Department of Mathematical Informatics, Graduate School of Information Science and Technology, The University of Tokyo, 7-3-1 Hongo, Bunkyo-ku 113-8656, Japan; 2 The Department of General Systems Studies, Graduate School of Arts and Sciences, The University of Tokyo, 3-8-1 Komaba, Meguro-ku 153-8902, Japan

**Keywords:** minimum description length, non-negative tensor factorization, model selection, normalized maximum likelihood code length

## Abstract

Non-negative tensor factorization (NTF) is a widely used multi-way analysis approach that factorizes a high-order non-negative data tensor into several non-negative factor matrices. In NTF, the non-negative rank has to be predetermined to specify the model and it greatly influences the factorized matrices. However, its value is conventionally determined by specialists’ insights or trial and error. This paper proposes a novel rank selection criterion for NTF on the basis of the minimum description length (MDL) principle. Our methodology is unique in that (1) we apply the MDL principle on *tensor slices* to overcome a problem caused by the imbalance between the number of elements in a data tensor and that in factor matrices, and (2) we employ the *normalized maximum likelihood* (NML) code-length for histogram densities. We employ synthetic and real data to empirically demonstrate that our method outperforms other criteria in terms of accuracies for estimating true ranks and for completing missing values. We further show that our method can produce ranks suitable for knowledge discovery.

## 1. Introduction

### 1.1. Motivation

In recent years, as the variety of data has grown rapidly, the data often contains more than two attributes and there are only non-negative values. For example, purchase data can be constructed as trading amounts of customers × commodities × shops × times. Another example is that web access data can be organized as access times of hosts × users × months. Such data can be conveniently organized as a non-negative high dimensional data tensor and analyzed by means of Non-negative Tensor Factorization (We focus on non-negative CANDECOMP/PARAFAC (CP) decomposition [1] in this paper) (NTF, [2]), which can be thought of as a generalization of well-known methodology of Non-negative Matrix Factorization (NMF, [3]).

For a third-order non-negative data tensor $X\in R_{+}^{I\times J\times K}$ and a non-negative rank R∈N, NTF factorizes X into three factor matrices $T\in R_{+}^{I\times R}$, $U\in R_{+}^{J\times R}$, and $V\in R_{+}^{K\times R}$. Denoting $x_{ijk}$, $t_{ir}$, $u_{jr}$ and $v_{kr}$ as the element of X,T,U and *V* respectively, the element-wise approximate factorization of the NTF model can be formulated as follows:

$$x_{ijk}=\sum_{r=1}^{R}t_{ir}u_{jr}v_{kr}+e_{ijk}.$$

Here, $e_{ijk}$ represents an element in the approximation error tensor $E\in R^{I\times J\times K}$. For simplicity, we denote the non-negative rank as rank in the rest of this paper following the terminology in [4]. Note that this is not the same notion as rank in matrices.

In contrast to NMF, NTF can not only extract the patterns hidden in the data tensor, but also simultaneously analyze multiple factors. For example, in the web access log analysis, three factorized matrices can represent influences of hosts, users and months, in which NTF can analyze not only users’ web behaviors but also its change according to time. As a method of multi-way analysis, NTF has been widely applied to various fields such as signal representations [5], sound source analysis [6] and computer vision [4].

There is a problem that we have to determine the value of *R* before performing NTF on a data tensor, which will greatly influence the factorized result. In fact, a successful decision of rank *R* can lead to an effective factorization which extracts vital features as well as eliminates the noise in the data. On the contrary, if the selected rank is too small, NTF will fail to reel off some key features, and if it is too large, we may obtain a result that is much influenced by irregularities of data.

Even though the selection of rank is very important, in most studies on NTF the value of *R* is simply determined by trial and error or specialists’ insights, and there does not exist a good way to determine *R* automatically.

### 1.2. Contribution

The contribution of this paper is summarized as follows:

(A) *Proposal of a novel criterion for rank estimation for NTF.* In this paper, we propose a method to select an appropriate value of rank for NTF based on the *minimum description length (MDL) principle* [7]. The MDL principle asserts that the best model must have the minimum code-length to encode the data as well as the model. The reasons why we choose the MDL principle among a number of existing statistical model selection criteria (e.g., AIC [8], BIC [9], cross-validation (CV), etc.) are: (1) it has consistency [10], and (2) it is guaranteed rapid convergence in the framework of probably approximately correct (PAC) learning [11].

Our application of the MDL is not straightforward. It is unique in the following two aspects:

(A-a) *Tensor slice based approach:* It is not appropriate to employ the MDL principle directly to NTF because the number of elements in factor matrices is too much smaller than that in a data tensor, hence the model complexity might be ignored inappropriately. We call this problem the *imbalance problem*. Our key idea to resolve this problem is that we first slice the data tensor into multiple *tensor slices*, namely matrices, then select the rank for each tensor slice, and finally select the largest rank of tensor slices as the best rank of the data tensor. We call this methodology the *tensor slice based approach*.
(A-b) *NML-based code-length calculation:* When the goodness of a model is measured in terms of its code-length, the selection of a coding method is important. We specifically utilize the *normalized maximum likelihood (NML) code-lengths* [12] to encode the results of factorization. This is due to the facts (1) the NML code-length has optimality under the Shtarkov’s minimax regret [13], and (2) it achieves Rissanen’s lower bound on code-length [10].

(B) *Empirical demonstration of effectiveness of the proposed criterion.* We employ synthetic and real data sets to empirically demonstrate the effectiveness of our proposed method in terms of:

(B-a) *Accuracy for estimating true rank:* When the true rank that generates the data is known, we measure the goodness of any rank estimation method in terms of its estimation accuracy.
(B-b) *Accuracy for completing missing values:* When the true rank is unknown, we measure the goodness of any rank estimation method in terms of accuracy of competing missing values.

We compare the proposed method with other criteria such as AIC, BIC, CV in terms of both measures (B-a) and (B-b). We further show using real data sets of web access data and app usage data that our method actually produces suitable ranks so that knowledge discovery can be conducted by looking at the bases in combination with external knowledge.

The next section introduces some related work on rank selection criteria for NTF and NMF. Then, we propose our MDL-based method in Section 3, and presents a comparison method named MDL2stage in Section 4. In Section 5, we show experimental results of our proposed method and other criteria on both synthetic and real data sets to demonstrate the effectiveness of our method. Finally, we summarize the conclusion of this paper in Section 6.

## 2. Related Work

In most of practical studies on NTF, the value of rank is either decided by trial and error or by specialists’ insights (see e.g., [6,14]). In addition, AIC and BIC have also been used for model selection in NTF [15].

Unlike NTF, there are a number of rank selection methods for NMF. As a special case of NTF, NMF aims to factorize a non-negative data matrix $X\in R_{+}^{I\times J}$ into factor matrices $W\in R_{+}^{I\times R}$ and $H\in R_{+}^{R\times J}$ as X=WH+E, where *E* is the approximation error matrix. In addition to general methods such as AIC, BIC and cross-validation [16], more involved criterion have been developed to select the rank for NMF, such as the MDL criterion with latent variable completion [17] and the non-parametric Bayes method [18]. The MDL code-length under the assumption of model regularity is studied in [19].

Squires et al. [20] proposed two rank selection methods for NMF based on the MDL principle. In this study, data matrix *X* is considered as the message from a transmitter to a receiver and it is sent in such a way that a transmitter send *W*, *H* and *E* and a receiver reconstruct *X* from them. When rank *R* is low, encoding of *W* and *H* requires shorter code-lengths while that of *E* does longer one. On the contrary, when *R* is high, encoding of *W* and *H* requires longer code-lengths while that of *E* does shorter one. The MDL principle is used to find the best solution of the trade-off between the accuracy and the complexity. Squires et al. proposed two methods for calculating code-lengths with Shannon information: −logp(x) where the probability *p* is known in advance. Note that the NML code-length we use can be thought of as an extension of Shannon information into the situation where the parameter θ of probability distribution p(x|θ) is unknown in advance [10].

## 3. Proposed Method

This section proposes an MDL-based rank estimation method for NTF. To do this, we extend the study on NMF by [20] into NTF in a non-trivial way. As noted in Section 1.2, in the case of tensors, we may suffer from the imbalance problem, that is, the number of elements in factor matrices is too much smaller than that of elements in a data tensor. For instance, in case of I=J=K=50 and rank R=20, error tensor E has 50×50×50 = 125,000 elements, while the total number of elements in three factor matrices T,U,V is only 20×(50+50+50)=3000. In such cases, compared with the code-length for the error tensor, those of factor matrices are too small to have an influence on the total result. Therefore, the NTF-based rank selection methods tend to choose the model that best fits the data and ignore the complexity of model in some way. As a consequence, the trade-off between complexity and errors cannot be well formalized.

Our key idea is to take the tensor slice based approach. The overall flow of our method is summarized as follows: We first produce a number of tensor slices from a non-negative data tensor, then consider those slices as non-negative data matrices and employ NMF to factorize them. Next, we select a rank for each tensor slice so that the total code-length is minimized, and finally select the largest one among the selected ranks for slices as the rank of the original tensor. Note that we calculate the code-length with the *NML code-length*, rather than Shannon information used in [20].

First of all, for a third-order non-negative tensor $X\in R_{+}^{I\times J\times K}$, the three kinds of its two-dimensional slices are: Horizontal slices $X_{i}\in R_{+}^{J\times K}\left(i=1,...,I\right)$, lateral slices $X_{j}\in R_{+}^{I\times K}\left(j=1,...,J\right)$, and frontal slices $X_{k}\in R_{+}^{I\times J}\left(k=1,...,K\right)$. Each tensor slice can be treated as a matrix to be factorized as follows:

(1) $$X_{a}=W_{a,R}H_{a,R}+E_{a,R},$$

where $X_{a}$ represents a tensor slice of the non-negative data tensor X. $W_{a,R}$ and $H_{a,R}$ are the two non-negative factor matrices, both of which consist of *R* factors. $E_{a,R}$ denotes the error matrix. When the data tensor has a true rank *R*, any $X_{a}$ has the rank no more than *R*. Therefore, we could select appropriate ranks for all $X_{a}$s and select the maximum value among them as the rank of the tensor.

Next, we select a rank of $X_{a}$ so that the total code-length is minimum. The total code-length of $X_{a}$ with rank *R* is given by:

(2) $$L_{\mathrm{total}}\left(X_{a},R\right)=L\left(W_{a,R}\right)+L\left(H_{a,R}\right)+L\left(E_{a,R}\right),$$

where L(x) is the code-length required for encoding *x* under the prefix condition. In order to calculate the code-lengths for elements in $W_{a,R},H_{a,R},$ and $E_{a,r}$, we need to discretize them with an appropriate precision δ, since they are real-valued numbers with unlimited precision, which cannot be encoded. For a given precision δ, the elements in each matrix are first vectorized to build a vector, then discretized into bins with precision δ to create a histogram. Letting the minimum and maximum of elements be $v_{min}$ and $v_{max}$, respectively, the histogram has bins:

$$\begin{array}{cc} C_{i} & =\left[v_{min}+\left(i-1\right)\delta ,v_{min}+i\delta \right)\;\left(i=1,...,s-1\right),\\ C_{s} & =\left[v_{min}+\left(s-1\right)\delta ,v_{max}\right], \end{array}$$

where $s=\lceil (v_{max}-v_{min})/\delta \rceil$ is the number of bins.

Then we employ the NML code-length in order to compute the code-length of $x^{n}$, each of which denotes an element assigned to a bin. The NML code-length is a theoretically reasonable coding method when the parameter value is unknown. Let $p(x^{n}|\theta ,M)$ be the probability distribution with parameter θ under the model M. According to [12], given a data sequence $x^{n}$, the NML code-length $L_{\mathrm{NML}}\left(x^{n}|M\right)$ for $x^{n}$ under the model M is given by:

(3) $$\begin{array}{cc} L_{\mathrm{NML}}\left(x^{n}|M\right)= & -\log p\left(x^{n}|\hat{\theta }\left(x^{n}\right),M\right)+\log\int p\left(y^{n}|\hat{\theta }\left(y^{n}\right),M\right)dy^{n}, \end{array}$$

where $\hat{\theta }\left(x^{n}\right)$ denotes the maximum likelihood estimator of θ from $x^{n}$. The second term in Equation (3) is generally difficult to calculate. Rissanen [12] derived its asymptotic approximation formula as follows:

(4) $$\begin{array}{c} \log\int p\left(y^{n}|\hat{\theta }\left(y^{n}\right),M\right)dy^{n}=\frac{k}{2}\log\frac{n}{2\pi }+\log\int \sqrt{\left|I\left(\theta \right)\right|}d\theta +o\left(1\right), \end{array}$$

where *k* is the number of parameters in model M, $\left|I\left(\theta \right)\right|$ denotes the determinant of the Fisher information matrix, and $o\left(1\right)$ satisfies $\lim_{n\to \infty }o\left(1\right)=0$ uniformly over $x^{n}$.

For $W_{a,R}$ and $H_{a,R}$, the zero terms in factor matrices are generally much more than the non-zero terms. Thus we separately compute the code-length of zero terms, namely the first bin in the histogram, and non-zero terms as follows:

$$L\left(Y_{a,R}\right)=L_{\mathrm{zero}}\left(Y_{a,R}^{0}\right)+L_{\mathrm{NML}}\left(Y_{a,R}^{+}\right),$$

where $Y_{a,R}$ is either $W_{a,R}$ or $H_{a,R}$. $Y_{a,R}^{0}$ represents the zero terms in $Y_{a,R}$, and $Y_{a,R}^{+}$ denotes the non-zero terms in $Y_{a,R}$.

For the zero terms or the first bin in the histogram, by applying Equations (3) and (4) into the Bernoulli model, their NML code-length is calculated as follows:

(5) $$L_{\mathrm{zero}}\left(\cdot \right)=-n_{0}\log\frac{n_{0}}{n}-\left(n-n_{0}\right)\log\frac{n-n_{0}}{n}+\frac{1}{2}\log\frac{n\pi }{2},$$

where *n* is the total number of elements in $W_{a,R}$ or $H_{a,R}$, and $n_{0}$ denotes the number of zero values in this matrix.

For the binned data in $W_{a,R}^{+}$, $H_{a,R}^{+}$ and $E_{a,R}$, by applying Equations (3) and (4) into histogram densities with *s* bins, we can compute their NML code-lengths as:

(6) $$\begin{array}{cc} L_{\mathrm{NML}}\left(\cdot \right)= & -\sum_{i=1}^{s}n_{i}\log\frac{n_{i}}{n}+\frac{s-1}{2}\log\frac{n}{2\pi }+\log\frac{\pi^{s/2}}{\Gamma \left(s/2\right)}+\log n+L_{\mathrm{int}}\left(s-1\right), \end{array}$$

where $n_{i}$ is the number of elements in the *i*-th bin, and $\Gamma \left(\cdot \right)$ is the gamma function. $L_{\mathrm{int}}\left(s-1\right)$ is the code-length of an integer [10], which can be computed as:

(7) $$L_{\mathrm{int}}\left(y\right)=\log2.865+\log y+\log\log y+...,$$

where the summation is taken over all the positive iterates. Using Equations (5) and (6), the total description length of $X_{a}$ can be calculated as follows:

(8) $$\begin{array}{c} L_{\mathrm{total}}\left(X_{a},R\right)=L_{\mathrm{zero}}\left(W_{a,R}^{0}\right)+L_{\mathrm{NML}}\left(W_{a,R}^{+}\right)+L_{\mathrm{zero}}\left(H_{a,R}^{0}\right)+L_{\mathrm{NML}}\left(H_{a,R}^{+}\right)+L_{\mathrm{NML}}\left(E_{a,R}\right). \end{array}$$

After we apply the MDL principle to select the rank with the shortest total code-length for each tensor slice, we select the largest one from all of slices’ ranks as the rank of the tensor. This estimate can be seen as an lower bound of the rank of the tensor from the fact that the ranks of slices is smaller than that of the data tensor, that is, if the rank of tensor *X* is *R* and the decomposition is represented as $x_{ijk}=\sum_{r=1}^{R}t_{ir}u_{jr}v_{kr}$, each slice $X_{a}$ can be represented as $W_{a}^{\prime }H_{a}^{\prime }$ with rank *R* matrices $W_{a}^{\prime }$ and $H_{a}^{\prime }$. For example, each element of $X_{i}={\left(x_{ijk}\right)}_{jk}$ can be represented as $x_{ijk}=\sum_{r=1}^{R}w_{jr}h_{kr}$, where $w_{jr}=t_{ir}u_{jr}$ and $h_{kr}=v_{kr}$.

We show the entire procedure in Algorithm 1.

**Algorithm 1** Rank Selection with Tensor Slices
 Slice a non-negative third-order data tensor $X\in R_{+}^{I\times J\times K}$ into tensor slices $X_{a}$   **for** a=1toI+J+K **do**  **for** $R=1\;\mathrm{to}\;\min\left(I,J,K\right)$ **do**   Perform NMF on $X_{a}$ to obtain $W_{a,R}$ and $H_{a,R}$    Calculate $E_{a,R}=X_{a}-W_{a,R}H_{a,R}$    Compute the total code-length using Equation (8)    **end for**   Select the rank of a tensor slice: 
$$R_{slice}\left(X_{a}\right)=\underset{R}{\mathrm{argmin}}L_{total}\left(X_{a},R\right).$$
 **end for**  Select the rank of tensor: 
$$R_{tensor}=\max R_{slice}\left(X_{a}\right).$$
  **return** $R_{tensor}$


## 4. Comparison Method: MDL2stage

We further developed a novel algorithm, MDL2stage, as a comparison method. MDL2stage is based on tensor slice similarly with the proposed method, but it encodes the factorized results of NMF via the two-stage code-length. All of its calculation is exactly the same as our proposed method except the way of encoding the error matrix and the non-zero terms in the factor matrices.

In MDL2stage, we fit a parametric probability distribution to the histogram to estimate the probability density of each bin. Generally, we assume that elements in the histogram generated by the error matrix $E_{a,R}$ follow the normal distribution, and assume that the non-zero elements in the histograms of two factor matrices $W_{a,R}$ and $H_{a,R}$ are gamma-distributed. Then we use two-stage code-length to calculate the description length of $E_{a,R}$ and the non-zero terms in $W_{a,R}$ and $H_{a,R}$ as follows:

(9) $$\begin{array}{cc} L_{2-stage}\left(Y_{a,R}^{+}\right) & =-\sum_{i=1}^{s}n_{i}\log\frac{\rho_{i}}{\sum_{i=1}^{s}\rho_{i}}+\log\left(n-n_{0}\right)+\log n+L_{\mathrm{int}}\left(s-1\right), \end{array}$$

where $Y_{a,R}^{+}$ is $E_{a,R}$ or non-zero terms in $W_{a,R}$ or $H_{a,R}$, $\rho_{i}$ denotes the estimated probability of an element to be in the *i*-th bin.

The total code-length for a tensor slice $X_{a}$ with rank *R* is:

$$L_{\mathrm{total}}\left(X_{a},R\right)=L_{\mathrm{zero}}\left(W_{a,R}^{0}\right)+L_{2-stage}\left(W_{a,R}^{+}\right)+L_{\mathrm{zero}}\left(H_{a,R}^{0}\right)+L_{2-stage}\left(H_{a,R}^{+}\right)+L_{2-stage}\left(E_{a,R}\right).$$

Again, we apply the MDL principle to choose the rank with the shortest total code-length for each slice, and select the greatest rank from all of slices’ ranks as the rank of tensor.

As for the computational complexity for our proposed method and MDL2stage, the cost of factorizing a tensor slice with size I×J is $O\left(IJR\right)$ for each iteration. Since there are *K* such tensor slices, the total computational complexity of the two methods based on tensor slice is $O\left(IJKR\right)$ per iteration. By similar arguments, performing NMF on all I+J+K tensor slices costs $O\left(IJKRT_{NMF}\right)$, where $T_{NMF}$ denotes the number of iterations in NMF.

Although theoretically we have to perform NMF on I+J+K slices, numerical experiments have proven that actually we only need to factorize *K* tensor slices which have the biggest size I×J, where we assume that *K* is the smallest value among *I*, *J* and *K*. This is because that smaller tensor slices usually give comparatively low ranks. Therefore, when we choose the largest rank over all ranks of slices, whether or not to use tensor slices with smaller sizes does not influence the final result.

## 5. Experiments

### 5.1. Comparison Methods

In addition to our proposed method and MDL2stage, we employ other five criteria for comparison. They are categorized into two classes: One is the based on tensor slice and the other is based on tensor.

For tensor slice based criteria, we first slice the data tensor into tensor slices $X_{a}$, then perform NMF on the sliced matrices to obtain factor matrices $W_{a,R}$ and $H_{a,R}$, and the error matrix $E_{a,R}$. There are four comparison methods based on tensor slice. Two of them are derived from AIC (Akaike’s information criteria) [8], which we denote as AIC1 and AIC2. In order to apply AIC, we consider two probabilistic models $p\left(X_{a}|W_{a,R},H_{a,R},\theta_{E}\right)$ where $X_{a}$ is generated by $X_{a}=W_{a,R}H_{a,R}+E_{a,R}$, and elements in $E_{a,R}$ follow the histogram model in our proposed method and MDL2stage, respectively. Therefore, the leading terms in AIC1 and AIC2 are the same as those in Equations (6) and (9) for $E_{a,R}$, but the remaining penalty terms are both $R\left(I+J\right)+s$. The other two tensor slice based criteria are derived from BIC (Bayesian information criteria) [9], which we denote as BIC1 and BIC2. The leading terms in them are the same as those in AIC1 and AIC2, but the remaining penalty terms are replaced with $\left(\left(R\left(I+J\right)+s\right)/2\right)\log n$, where *n* is the number of elements in $E_{a,R}$. We select the rank which minimizes those criteria as the tensor slice’s rank, and choose the greatest one from all of the slice’s ranks as the rank of the tensor.

The only tensor-based method is cross-validation, it leaves out some elements from the tensor and then performs NTF to factorize the tensor with missing cells. It then calculates the prediction error between the predicted values of the left out elements and their original values. Therefore, the rank with the lowest prediction error is chosen as the rank of the tensor.

We made the implementation of our proposed method and all of the comparison methods except cross validation available (https://github.com/fuyunhui/Model-Selection-for-NTF).

### 5.2. Experiments on Synthetic Data

We first evaluate our method in terms of accuracy for estimating true ranks using synthetic data. In this experiment, we employed SimTensor [21] to generate synthetic data tensors. To create a third-order non-negative artificial tensor $X\in R_{+}^{I\times J\times K}$, we used the “stochastic” module to generate the random factor matrices $T\in R_{+}^{I\times R^{*}}$, $U\in R_{+}^{J\times R^{*}}$, and $V\in R_{+}^{K\times R^{*}}$ with a given true rank $R^{*}$, then the synthetic tensor was obtained by computing the sum of multi-way outer products. We created several non-negative artificial tensors with different true rank $R^{*}$ and the same size 2000×1000×5. All of the synthetic tensors were applied 1% sparse white noise, and the true rank $R^{*}$ was set from 15 to 40, which were comparatively low because we want to evaluate the performance of each rank selection method accurately.

As we knew the true value of rank for synthetic data tensors, following studies in [17], the performance of any rank selection method can be evaluated by its benefit value. *Benefit* measures how close the predicted rank is to the true rank. The definition of benefit is given as follows:

$$B\left(R^{*},T\right)=\frac{1}{T}\sum_{t=1}^{T}\max\left(0,1-\frac{|R^{*}-R_{tensor}^{t}|}{U}\right),$$

where *T* denotes the number of trials, which was set to be 10 in this experiment, and $R_{tensor}^{t}$ represents the estimated rank in the *t*-th trial. If $|R^{*}-R_{tensor}^{t}|>U$, then the benefit becomes zero. We set U=10 and precision δ=0.001.

The benefits for seven criteria versus six different values of true rank are shown in Table 1. Bold numbers indicate the best result for each true rank. Obviously, when the true rank $R^{*}$ was very small compared with the size of the tensor, cross-validation gave a good result, but as $R^{*}$ increased, the rank selected by our proposed method was closest to $R^{*}$.

The reason that our method performed low benefits in case of small true rank can be considered as follows. When the true rank is extremely small and the noise is relatively large compared with the Frobenius norm of the data tensor X, the code-length of error tensor becomes to dominate the entire code-length even when the true rank is given. In such cases, methods based on the MDL principle such as our methods and MDL2stage leads to overestimate the true rank.

In order to demonstrate argumentation above, we performed additional experiments with smaller noises. Using 0.5% sparse white noise instead of 1% sparse noise, we evaluated the benefit of the proposed method and MDL2stage in case of $R^{*}=15,20$. The proposed method exhibited 0.82 and 0.89 of benefit for $R^{*}=15$ and 20, whereas MDL2stage exhibited 0.69 and 0.62. Therefore, we conclude that the reason that the proposed method underperformed when the true rank is extremely small is because the noise is relatively large to the size of data tensor. Moreover, we can see MDL2stage tends to underestimate the true rank and these effects have canceled out in this experiments of extremely small true rank with large noise.

Note that the computational complexity of cross-validation is $O\left(tIJKR\right)$ where *t* is the number of repeating leaving out missing cells and summing the prediction error. Thus cross-validation will take too much time for large tensors and high candidate ranks. Moreover, AIC1 and AIC2 tended to choose more than ten larger rank than $R^{*}$, so their benefits were always 0. Thus, we have demonstrated that our proposed method is able to choose a suitable rank for large synthetic data tensors with a comparatively small but not extremely small true rank. We also experimentally confirmed that, in spite of the fact that our estimate is based on the lower bound of the tensor rank, our proposed method does not suffer terribly from underestimation.

### 5.3. Experiments on Real Data

#### 5.3.1. On-Line Retail Data Set

The on-line retail data set [22] has been published by UCI machine learning repository [23]. It consists of transnational logs for transactions occurring between 12/2010 and 12/2011 for an on-line retail. We constructed a third-order non-negative data tensor X containing 911 commodities, 671 customers and 12 months. So an element $x_{ijk}$ indicated the monthly trading amount of the *i*-th commodity bought by the *j*-th customer in the *k*-th month.

Using this data set, we evaluated the performance of our method. The evaluation metric was the *accuracy of completing missing values* since the true rank was unknown in this case. This accuracy was measured as follows: We first estimated the rank of tensor with different rank selection criteria, then left out some missing cells from the data tensor and conducted cross-validation to compute the prediction error of missing values for the selected ranks. We repeated this process ten times by randomly selecting 5% missing cells to calculate the average prediction errors for all the methods. We compared our method with other methods, except cross-validation. This is because the computation cost of cross-validation is too high especially for tensors with large size and a high rank. The precision δ was set to be 0.001.

Table 2 shows ranks estimated by the six criteria and their prediction errors. AVE *R* and STDDEV mean the average value of estimated ranks and its standard deviation through ten trials, respectively. Prediction error for the selected rank is denoted as Error. We see that our proposed method obtained the lowest prediction error. The value of prediction errors seems to be large. Note, however, that the squared Frobenius norm of the data tensor X is $1.18\times 10^{6}$. So we could say that the prediction error of our proposed method was a suitable value. In addition, the number of patterns chosen by our method was 37 in average, which might be a sensible choice for 911 commodities and 671 customers, especially compared with 7 for BIC-based criteria and 500 for MDL2stage and the AIC-based criteria.

#### 5.3.2. Web Access Data Set

We also evaluate the performance of our method in terms of *knowledge discovery*. We employed the data set containing web access logs during 6 months provided by VALUES, Inc. (https://www.valuesccg.com/). Those web access logs were organized into a third-order data tensor X. The three axes of X were user, host and time period. So an element $x_{ijk}$ represented the count of user *i* accessed host *j* during time period *k*, and the size of X was 2354×2131×24. Here, we split a week into weekdays and weekends, and split a day into 12 time periods, so there were 24 time periods in total.

We make the following process for knowledge discovery.

**Step 1:***Conduct NTF to extract bases.* We conduct NTF with rank *R* estimated by a criteria. The factor matrix corresponding to the axis of user can be considered as *R* patterns of users.

**Step 2:***Specify a pattern of profiles and time periods.* We are given external knowledge about users’ profiles (e.g., age, gender, occupation) in addition to the data tensor. Thus we specify a pattern of combinations of profile and time period such as “female & daytime” that we like to investigate.

**Step 3:***Give matching scores to each base.* For each base obtained in Step 1, we give it a score to indicate how well the user and period in this base match the pattern specified in Step 2. The score is calculated as follows: First compute a matching score of a user belonging to a base, then multiply the score by the user’s weight in the base, finally sum all the matching scores up to get the total score. The matching score is calculated as +1 or more if the attribute matches, −1 or less otherwise.

**Step 4:***Knowledge discovery from the base with the largest score.* Once scores are calculated for all the bases, the base that best matches the pattern specified in Step 2 can be found. We can characterize the pattern by analyzing such a base.

In addition, we generated random vectors and specified patterns for user and time period as baselines. We set precision δ=0.0001, and we repeated each experiment 10 times.

Table 3 shows ranks estimated by six criteria and their scores for four specified patterns. The bottom 2 rows show the baselines, and bold numbers indicate results having the largest scores. We observed that our proposed method achieved best scores for all the specified patterns except “male at day”. Therefore we consider our proposed method selected most suitable ranks whereas AIC-based methods and MDL2stage preferred larger ranks, and BIC-based methods inclined to smaller ranks. For the “male at day” pattern, we checked the highest scored base with rank R=195 and found that its time period weights had a non-zero value only at one point, which led to a high score although it is not reasonable as a meaningful web access pattern.

**Figure 1:**
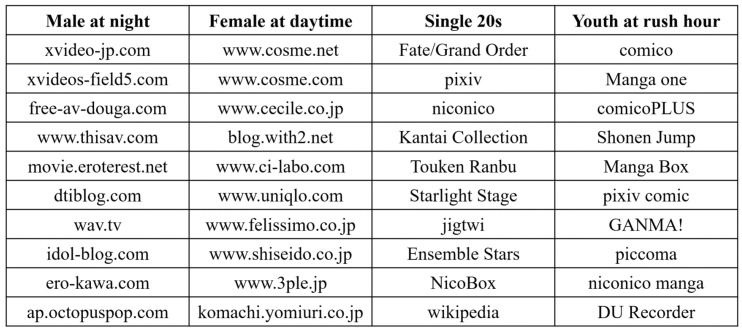
The top 10 valued web pages and apps in 4 winner bases.

The left two columns in Figure 1 show the top 10 highest valued web pages in bases with the highest scores for “male at night” pattern and “female at day” pattern, respectively. It is clearly observed that all of the top 10 valued websites in the first pattern were related to adult video, while those in the second pattern were mainly related to cosmetic and fashion. Actually, most of the top 100 highest valued users in the “male at night” pattern were men who lived in Kanto area in Japan with ages from 35 to 54 years old, and the access time was distributed only at midnight. In addiction, it turned out that most of the top 100 highest valued users in “female at day” pattern were middle-aged women, and the access time was distributed from 8 a.m. to 8 p.m. For simplicity and the limitation of paper length, we do not show the top 10 highest valued web pages for “male at day” pattern and “female at night” pattern. Thus, we observe that our proposed method produced a reasonable rank while extracting bases better matching the specified patterns.

#### 5.3.3. App Usage Data Set

We employed the data set consisting of app usage logs during 1 year provided by VALUES, Inc. and applied the same analysis as the web access data. We constructed a third-order data tensor X containing 2593 users, 2118 apps and 12 time periods of each day. Then an element $x_{ijk}$ denotes the count of user *i* used app *j* during time period *k*. We set precision δ=0.0001, and repeated each trial 10 times to calculate the average rank and its standard deviation.

Table 4 shows ranks estimated by six criteria and their scores.This time, our proposed method found best bases for both two specified patterns. The right two columns in Figure 1 show the top ten highest valued apps in bases with the highest scores. For the “single 20s” pattern, most of the top ten highest valued apps are popular smart phone games that target the crowd who love animation and comics. For the “Youth at rush hour” pattern, all of the top ten highest valued apps except the last one belong to the comic category in Google Play because in Japan, we can find office worker using their smart phone to watch comics at rush hour everywhere. Thus, we observe that our proposed method selected more suitable rank than other criteria for the app usage data set as well.

## 6. Conclusions

This paper has proposed a novel rank selection method for NTF on the basis of the MDL principle. Our method is unique in the tensor slice approach and the NML-based code-length calculation. Using synthetic and real data sets, we have demonstrated the effectiveness of our proposed method in terms of accuracies for estimating true rank and completing missing values. The proposed method has turned out to perform well, especially when the size of the data tensor is large and the true rank is not extremely small. We have also shown that our method produces suitable ranks for knowledge discovery. Future work can include the development of more computationally efficient variants of our method. Recently the decomposed normalized maximum likelihood (DNML) codelength criterion [24] has been developed for latent variable modeling on the basis of the MDL principle. It has been verified that DNML is widely applicable and has good theoretical properties. It remains for future study how we can apply DNML to the problem of tensor rank estimation.

## Figures and Tables

**Table 1:** Benefits for seven criteria versus six different values of true rank. Boldfaces describe best performances.

True Rank	15	20	25	30	35	40
Proposed	0.11	0.45	**0.94**	**0.88**	**0.81**	**0.92**
MDL2stage	0.92	0.74	0.61	0.47	0.37	0.28
AIC1	0	0	0	0	0	0
AIC2	0	0	0	0	0	0
BIC1	0.54	0.72	0.87	0.76	0.63	0.72
BIC2	0.84	0.66	0.4	0.29	0.1	0.08
CV	**0.95**	**0.86**	0.73	0.65	0.62	0.71

**Table 2:** Ranks estimated by six criteria and their prediction errors. Boldfaces describe best performances.

Rank and Error	AVE *R*	STDDEV	Error
Proposed	**36.8**	**2.39**	$4.99\times 10^{4}$
MDL2stage	548.1	23.26	36.52 × $10^{4}$
AIC1	472	145.26	39.38 × $10^{4}$
AIC2	546.1	22.09	38.89 × $10^{4}$
BIC1	7.2	1.55	5.27 × $10^{4}$
BIC2	3	0	5.42 × $10^{4}$

**Table 3:** Rank estimated by 6 criteria and their scores for web data. Boldfaces describe best performances.

Rank and Score	AVE *R*	STDDEV	Male at Night	Female at Day	Male at Day	Female at Night
Proposed	**82.4**	**15.23**	**0.642**	**0.823**	0.685	**0.947**
MDL2stage	194.4	25.40	0.598	0.666	**0.745**	0.745
AIC1	155.9	21.05	0.612	0.683	0.564	0.607
AIC2	196.7	23.71	0.599	0.679	0.577	0.794
BIC1	13.9	1.52	0.160	0.197	0.140	0.201
BIC2	35.0	6.09	0.471	0.566	0.446	0.324
Randomly generated vectors			0.070	−0.022	0.004	−0.004
Specified generated patterns			0.365	0.298	0.302	0.256

**Table 4:** Rank estimated by 6 criteria and their scores for app data. Boldfaces describe best performances.

Rank and Score	AVE *R*	STDDEV	Single 20s	Youth at Rush Hour
Proposed	**61.8**	**10.25**	**0.990**	**0.849**
MDL2stage	147.6	22.27	0.939	0.722
AIC1	97.4	15.15	0.963	0.732
AIC2	148.5	19.72	0.939	0.723
BIC1	14.5	3.44	0.467	0.602
BIC2	26.7	8.93	0.617	0.686
Randomly generated vectors			−0.109	0.300
Specified generated patterns			0.498	0.655

## Abbreviations

The following abbreviations are used in this manuscript:

NTF	Non-negative Tensor Factorization
MDL	Minimum Description Length
NML	Normalized Maximum Likelihood
NMF	Non-negative Matrix Factorization
AIC	Akaike Information Criterion
BIC	Bayesian Information Criterion
CV	Cross Validation
